# Disentangling the role of different resting-state neural markers of adolescent behavioral inhibition and social anxiety

**DOI:** 10.1016/j.dcn.2025.101560

**Published:** 2025-04-24

**Authors:** Madison Politte-Corn, Sarah Myruski, Bridget Cahill, Koraly Pérez-Edgar, Kristin A. Buss

**Affiliations:** a The Pennsylvania State University, Department of Psychology, University Park, PA, USA; b The Pennsylvania State University, Department of Human Development and Family Studies, University Park, PA, USA

**Keywords:** EEG, Resting state, Adolescent, Social anxiety, Behavioral inhibition, Asymmetry, Delta-beta coupling

## Abstract

One of the most reliable predictors of adolescent social anxiety is the temperamental profile of behavioral inhibition (BI), but there is considerable heterogeneity in this association. Resting-state EEG-based neural markers, namely frontal alpha asymmetry and delta-beta coupling (DBC), hold promise for improving our understanding of the relation between BI and social anxiety symptoms during adolescence. The current study aimed to (1) clarify the relation between these neural markers, BI, and social anxiety and (2) examine the moderating role, individually, of frontal alpha asymmetry and DBC on the BI-social anxiety link. Participants were 97 adolescents (*M*_age_ = 14.29 years, *SD*_age_ = .98; 84.4 % White, 3.1 % Black, 12.5 % multiracial; 54.6 % female) and their parents. Parents reported on adolescent BI and adolescents self-reported social anxiety symptoms. Additionally, adolescents provided EEG data across a 6-minute resting task, from which measures of frontal alpha asymmetry and DBC were derived. Results indicated that stronger DBC was directly associated with higher social anxiety symptoms, but not BI, and did not moderate the association between BI and social anxiety. In contrast, frontal alpha asymmetry was not directly associated with either BI or social anxiety but interacted with BI to predict avoidance and distress to social situations, such that greater relative right activation predicted a stronger BI-social anxiety link. However, this effect did not survive correction for multiple comparisons. Findings suggest that high DBC may mark a general vulnerability for social anxiety symptoms, whereas frontal alpha asymmetry may potentiate the risk for social anxiety symptoms specifically among BI youth.

## 1 Introduction

Adolescence is marked by substantial social and neurobiological development, which coincides with an increased risk for social anxiety symptoms and disorder (Bitsko et al., 2022, Nelson et al., 2016). Internalizing difficulties, such as social anxiety symptoms, that peak during adolescence are associated with a particularly poor course compared to later-onset internalizing psychopathologies (Colman et al., 2007, Korhonen, 2018), which makes the early detection of risk critical. One of the most reliable, early emerging predictors of social anxiety is the temperamental profile of behavioral inhibition (BI), which is early appearing, relatively stable across development, and characterized by a heightened sensitivity to novelty, especially if social in nature (Gest, 1997, Kagan et al., 1984). Yet, only around 40 % of BI youth will develop social anxiety disorder (Clauss and Blackford, 2012), indicating substantial heterogeneity in this association and the presence of moderators that influence developmental outcomes.

The collective literature on the BI-social anxiety link implicates avoidance motivation and emotion regulation difficulties as biobehavioral processes that likely increase risk (Coplan et al., 2018, Degnan et al., 2014, Smith et al., 2019). These vulnerabilities can be indexed at the neural level through electroencephalogram (EEG)-derived oscillation patterns, namely frontal alpha asymmetry and delta-beta coupling (DBC; McManis et al., 2002; Schutter and Knyazev, 2012). These neural markers could potentially improve our understanding of which BI youth experience greater social anxiety symptoms during adolescence, but this question remains to be empirically tested and is the goal of the current study.

### 1.1 The association between behavioral inhibition and social anxiety

BI was first introduced to describe a heightened vigilance and lack of approach to novelty in toddlers (García-Coll et al., 1984, Kagan et al., 1984). Subsequent work delineated a developmental trajectory from early BI to social reticence and avoidance in childhood (Degnan et al., 2014, Kagan et al., 1988, Kagan, 1998), followed by the emergence of anxiety symptoms and disorders in adolescence (Chronis-Tuscano et al., 2009, Hirshfeld-Becker et al., 2007). Specifically, BI children are nearly four times more likely than their uninhibited peers to meet diagnostic criteria for social anxiety disorder (SAD; Chronis-Tuscano et al., 2009). Despite this robust association, less than half of BI youth (∼40 %) will develop SAD (Clauss and Blackford, 2012), and there is a need to improve the identification of which inhibited children are at greatest risk.

Avoidance motivation and difficulties with emotion regulation are two biobehavioral processes by which BI may confer risk for social anxiety symptoms (Buss, 2011, Degnan et al., 2014, Rubin et al., 2018, Smith et al., 2019). Both avoidance and emotion regulation difficulties are linked to excessive excitability of limbic brain regions, such as the amygdala, and manifest with heightened emotional reactivity and social fear (Blackford et al., 2011, Blackford et al., 2018, Fox et al., 2021, Fox et al., 2023, Kagan et al., 1999, Shackman et al., 2016). A robust literature has demonstrated that temperamentally fearful youth have difficulty regulating this heightened emotional arousal (Buss, 2011, Buss et al., 2018, Smith et al., 2019), which is achieved at the neural level through interactions between cortical and subcortical activity (Guyer et al., 2016, Schutter and Knyazev, 2012). Further, in social contexts, BI youth tend to remain on the periphery and avoid social interaction (Degnan et al., 2014, Henderson et al., 2018, Rubin et al., 2018), which is thought to impede the development of socio-cognitive skills and exacerbate avoidance motivation (Rubin et al., 2009). These pre-existing, and increasingly entrenched, vulnerabilities may become especially problematic during adolescence, when peer interactions take on increased importance. This difficulty with social contexts is exacerbated by normative developmental changes marked by greater emotional reactivity and arousal and difficulty with regulation in adolescence (Guyer et al., 2016, Nelson et al., 2016, Silvers et al., 2012).

### 1.2 Frontal alpha asymmetry and delta-beta coupling index relevant biobehavioral vulnerabilities

Emotion and motivation-related brain functions can be indexed by oscillatory patterns across various frequency bands derived from EEG, each of which have unique proposed functions and behavioral correlates (Engel et al., 2001, Knyazev and Slobodskaya, 2003, Schutter and Knyazev, 2012). EEG-derived metrics are advantageous because they are more economical than other neuroimaging techniques and can reveal social-emotional vulnerabilities not apparent from behavioral observations or self-report (Kujawa et al., 2014, Luck and Kappenman, 2011). Relative to task-based EEG measures such as event-related potentials, resting-state EEG metrics have the advantage of reflecting dispositional neural patterns rather than response to a specified stimulus and are more easily administered and compared across development. Although recorded at the scalp, these oscillations result from interactions between cortical and subcortical regions comprising complex neural networks (Schutter and Knyazev, 2012). Here, we focus on two EEG-derived markers previously associated with our core constructs of interest (Anaya et al., 2021a, Howarth et al., 2016, McManis et al., 2002, Poole et al., 2020).

Slow wave activity in the delta frequency range is thought to underlie subcortical brain functions, such as those related to limbic area activation, whereas fast wave activity in the alpha and beta frequency ranges reflects intra-cortical brain functions thought to be linked to cognitive processing and regulation (Engel et al., 2001, Knyazev and Slobodskaya, 2003, Schutter and Knyazev, 2012). Frontal alpha asymmetry captures relative levels of alpha activity across frontal electrodes. Delta-beta coupling, in turn, reflects the crosstalk between these frequency ranges, as reflected in correlation over time. Together, they provide unique insights into motivational and regulatory processes that may moderate the developmental transition from BI to social anxiety. Indeed, both frontal alpha asymmetry and delta-beta coupling predict the social behavior of BI children in a novel dyadic interaction (Anaya et al., 2021a).

#### 1.2.1 Frontal alpha asymmetry and avoidance bias

Frontal alpha asymmetry is a well-established marker of approach versus avoidance tendencies (Davidson, 1992, Davidson, 1998, Harmon-Jones and Gable, 2018). Alpha power is inversely related to activation, such that greater right activation is reflected by lower right alpha power. Greater right activation is thought to reflect avoidance or withdrawal tendencies, whereas greater left activation is thought to reflect approach tendencies (Davidson, 1992, Davidson, 1998, Harmon-Jones and Gable, 2018). Cross-sectional studies in infants and toddlers demonstrate that higher right EEG asymmetry is associated with shy/fearful temperament (Buss et al., 2003, Theall-Honey and Schmidt, 2006). Longitudinally, fear prospectively predicts greater relative right asymmetry across early childhood, but asymmetry did not predict fear (Howarth et al., 2016), and toddlers characterized as temperamentally fearful continue to display greater right frontal activation as adolescents (McManis et al., 2002).

However, some studies did not find a significant direct association between frontal alpha asymmetry assessed during adolescence and BI or social anxiety (Harrewijn et al., 2019). Rather, for adolescents with a history of BI, greater right frontal activity was associated with increased neural sensitivity to social threat, which in turn predicted greater social anxiety symptoms (Buzzell et al., 2017, Harrewijn et al., 2019). This is consistent with other work in adults showing that greater right frontal activation is associated with faster detection of threatening versus neutral faces and higher anxiety (Avram et al., 2010). Taken together, these studies suggest that avoidance bias, as indicated by greater relative right frontal asymmetry at rest, may develop as a compensatory strategy for some children with a fearful temperament or high threat sensitivity that developmentally confers risk for social anxiety symptoms.

#### 1.2.2 Delta-beta coupling and regulatory difficulties

Resting-state coupling across delta and beta frequency bands may provide a useful neurophysiological marker of emotion regulation vulnerabilities (Knyazev et al., 2006, Phelps et al., 2016, Venanzi et al., 2024). Stronger delta-beta coupling is thought to reflect greater coherence between limbic and cognitive control regions, often conceptualized as a neural pattern of inefficient top-down regulation or overcontrol (Myruski et al., 2024, Phelps et al., 2016, Schutter and Knyazev, 2012). For example, prior work indicates that higher delta-beta coupling is associated with fearful temperament (Phelps et al., 2016, Poole et al., 2020, Poole and Schmidt, 2020, Vanpeer, 2008) and higher social anxiety in children and adolescents (Anaya et al., 2021a, Myruski et al., 2024). Further, the strength of midfrontal delta-beta coupling can be increased by the administration of cortisol (Vanpeer, 2008), which is generally heightened in children with fearful temperament and anxiety (Kagan et al., 1987, Schiefelbein and Susman, 2006). Drawing from these findings, greater delta-beta coupling has been interpreted as underlying the overcontrolled nature of anxiety-related phenotypes, potentially reflecting difficulties regulating arousal in subcortical networks (Knyazev et al., 2006, Knyazev and Slobodskaya, 2003).

#### 1.2.3 Neurodevelopmental mechanisms underlying frontal alpha asymmetry and delta-beta coupling during adolescence

Basic research on the development of brain circuitry involved in emotion regulation provides some insight into the neural mechanisms underlying these EEG markers. Prior work has shown that subcortical regions (e.g., the amygdala) increase in volume across puberty (Goddings et al., 2014) and mature earlier than cortical regions involved in top-down regulation (Galvan et al., 2006, Mills et al., 2014). This heightened limbic activation can be indexed by delta-band activity and likely manifests as greater sensitivity to threat (Fu et al., 2017), which in turn relates to both pubertal development and greater right frontal activation (Heffer and Willoughby, 2020). The subsequent development of regulatory cortical connections suggests an increasing dominance of higher frequency bands such as beta across adolescence, reflecting improvements in top-down regulation. Taken together, these findings suggest a developmental pattern of increasing right frontal activation and delta-beta coupling across adolescence, respectively indexing increases in threat sensitivity and subsequent improvements in cortical regulation, although this has not been tested and there are likely individual differences in these trajectories. Ultimately, these normative changes in brain circuitry may exacerbate pre-existing biological dispositions for heightened limbic activity and cortical overcontrol, manifested as BI temperament (Auday and Pérez-Edgar, 2019, Fu et al., 2017), potentially leading to the emergence of social anxiety symptoms and disorder.

### 1.3 The present study

Disentangling the role of frontal alpha asymmetry and delta-beta coupling in the BI-social anxiety relation is important because they have distinct etiological implications. Indeed, prior work suggests that distinct forms of frontal cortex dysregulation may underlie heterogeneity in anxiety symptoms (Roberts and Mulvihill, 2024). Moreover, examining these associations specifically during adolescence is critical due to changing peer dynamics and a concurrent increased risk for social anxiety symptoms and disorder (Harrewijn et al., (n.d, Ostlund and Pérez-Edgar, 2023). Taken together, the extant literature suggests that frontal asymmetry and delta-beta coupling may improve the prediction of which BI youth are at greatest risk for social anxiety symptom development during adolescence. However, whether these neural markers reflect temperamental risk, a general vulnerability for social anxiety symptoms, or are moderators that indicate increased risk for social anxiety among BI youth is still an open question. In the present study, we address this gap by examining (1) the direct associations between these neural measures, BI, and social anxiety symptoms and (2) the moderating role of frontal alpha asymmetry and delta-beta coupling, separately, on the BI-social anxiety link.

We also sought to examine whether these neural indices show specificity in predicting different symptom presentations. For example, greater relative right asymmetry may be more relevant for predicting avoidance behaviors associated with social anxiety symptoms rather than broadly characterizing social anxiety, given that alpha asymmetry is a well-established marker of avoidance versus approach tendencies (Davidson, 1998, McManis et al., 2002). Delta-beta coupling, however, may relate to social anxiety symptoms more broadly given links to emotion regulation difficulties (Knyazev et al., 2006, Myruski et al., 2024, Venanzi et al., 2024). Based on prior literature, we hypothesized that greater relative right frontal alpha asymmetry and higher delta-beta coupling at rest would strengthen the association between adolescent BI and social anxiety symptoms. Further, we hypothesized that alpha asymmetry would show specificity in predicting avoidance behaviors associated with social anxiety symptoms, whereas higher delta-beta coupling would predict social anxiety symptoms more broadly.

## 2 Method

### 2.1 Participants

Participants were 97 adolescents [53 (54.6 %) females; 44 (45.4 %) males] aged 13–17 (*M* = 14.29, *SD* = 0.98) drawn from a larger longitudinal study, which is described in detail elsewhere (Buss et al., 2023). Briefly, caregivers of 195 adolescents reported on adolescent temperament as part of a screening assessment. Subsequently, adolescents were invited to complete a series of remote questionnaires, clinical interviews, and an in-person EEG assessment annually for four years (T1, T2, T3, T4). T1 began when participants completed their first activity for the study (e.g., questionnaires), after which there was a 6-month window in which they could complete the EEG assessment for that timepoint. A similar window was in place for T2, which followed a year after T1. Due to the COVID-19 pandemic, in-person data collection was paused from March 2020 through August 2021, which resulted in a smaller EEG sample than anticipated. Consequently, we combined T1 and T2 to obtain a larger cross-sectional sample with greater power to detect the hypothesized effects, using data from the first timepoint (either T1 or T2) at which the EEG session was completed. Specifically, 80 participants provided EEG data at T1 (collected between May 2019 and July 2022), and an additional 17 participants missed the EEG assessment at T1 but provided EEG data at T2 (collected between October 2021 and September 2023). The significance and direction of all reported effects did not change when controlling for the timepoint at which data were collected.

Of the 195 participants who completed the screening assessment, 152 (77.95 %) provided questionnaire data and 97 (49.7 %) completed the in-person EEG assessment at either the first or second timepoint. Questionnaire and EEG data were contemporaneous (i.e., only data from the same timepoint were used). A sensitivity power analysis via Gpower 3.1.9.2 (Faul et al., 2007) indicated that a sample size of 97 was sufficient to detect effect sizes at and above *f*^*2*^ = 0.083 (*R*^2^ =.077) at 80 % power and two-tailed alpha level of.05 in a multiple regression model with three predictors. Participants who provided any EEG data did not differ from the larger study sample in the distribution of sex [χ^2^(1) = 2.31, *p* = .13], racial identity [χ^2^(2) = 0.16, *p* = .92], or familial income [χ^2^(15) = 19.53, *p* = .19], nor did this subset differ from the larger sample on BI [*t*(192) = 1.21, *p* = .23]. Participants who did not provide EEG data reported slightly higher social anxiety symptoms (*M* = 58.93; *SD* = 11.96) than participants who did provide EEG data [*M* = 54.52; *SD* = 13.14; *t*(143) = 2.02, *p* = .045]. Finally, participants who provided EEG data at the first versus second timepoint did not significantly differ by demographic characteristics or on the study variables (*p*s > .38), except for a marginally significant difference in sex distribution across timepoints [χ^2^(1) = 3.11, *p* = .078].

Parents reported their adolescent’s racial/ethnic identity and familial income. Of the 97 participants included in the final sample, 81 (84.4 %) were identified as White, 3 (3.1 %) were identified as Black or African American, 12 (12.5 %) were identified as more than one race, and one participant did not report their child’s racial identity. Five (5.2 %) participants further identified as Hispanic/Latinx. Of the 81 families from the final sample that provided complete data on annual household income, four (4.9 %) earned $30,000 or less, 25 (30.9 %) earned $31,000-$70,000, 16 (19.8 %) earned $71,000-$100,000, 23 (28.4 %) earned $101,000-$150,000, and 13 (16.0 %) earned more than $150,000. Pubertal development scores, assessed at the same timepoint as the EEG and averaged across parent and adolescent reports on a 4-point Likert Scale (*1* = not yet started; *2* = barely started; *3* = definitely started; *4* = seems complete), ranged from 1.20 to 4 (*M* = 2.82, *SD* = .69). Participants were recruited from central to south-central Pennsylvania regions, and all families provided informed consent/assent prior to participation. All study procedures were approved by the Institutional Review Board of Pennsylvania State University.

### 2.2 Measures

#### 2.2.1 Behavioral inhibition

BI was measured at screening using the Behavioral Inhibition Questionnaire (BIQ; Bishop et al., 2003), a parent-report questionnaire used to gauge child temperament characteristics particularly relating to shyness, fearfulness, and withdrawal. The questionnaire uses a 7-point Likert scale in which each numerical increase represents an increase in the frequency of observed behaviors. The overall BI score (30 items, *α* = 0.97) was used for the current study. The BIQ has previously been reliably used with older participants (Broeren and Muris, 2010).

#### 2.2.2 Social anxiety symptoms

Social anxiety was self-reported by adolescents using the Social Anxiety Scale for Adolescents (SAS-A; Greca and Lopez, 1998), a 22-item questionnaire used to evaluate fears and apprehensions regarding peers’ negative judgements and distress with new social situations. The SAS-A utilizes a 5-point Likert scale in which each numerical increase represents a stronger endorsement of behavior. The questionnaire is comprised of three subscales: Fear of Negative Evaluation (α = 0.95), Social Avoidance and Distress in New Situations (α = 0.90), and Social Avoidance and Distress- general (α = 0.77). The current study examined the total score (α = 0.95). We then assessed all three subscales individually as a follow-up exploratory analysis to probe for potential specificity in symptom presentation.

#### 2.2.3 EEG resting-state baseline

Adolescents were fitted with an actiCAP snap electrode cap (EASYCAP GmbH) with 32 channels and gel-based Ag/AgCl active sensors (Brain Products GmbH). Electrical activity from the electrodes were amplified through a BrainVision actiCHamp bioamplifer (Brain Products GmbH), then shown on a laptop screen in the testing room using Brain Vision Recorder acquisition software (Brain Products GmbH). During data collection, the high-pass filter was a single-pole RC filter with a 0.3 Hz cutoff (3 dB or half-power point) and 6 dB per octave roll-off. The low-pass filter was a two-pole Butterworth type with a 70 Hz cutoff (3 dB of half-power point) and 12 dB octave roll-off. Experimenters were trained to keep electrode impedances below 10 kΩ. However, data were analyzed if impedances were less than 20 kΩ, as active electrodes have circuitry at the electrode site designed to maintain good signal-to-noise ratio and permit recordings at high impedance (Kappenman and Luck, 2010). The EEG signal was transformed into digital data with a sampling rate of 1000 samples per second per channel. All electrode locations were referenced to FCz during recording.

Participants completed a 6-minute EEG resting state baseline. Experimenters kept time and instructed participants to open or close their eyes in an alternating fashion; eyes were kept open, facing a black computer monitor, for the first, third, and fifth minutes. The EEG data were divided into open or closed trials and then further into 1-second segments (360 total segments), then baseline corrected to the entire segment. Data were re-referenced to the mean of the right and left mastoid electrodes then filtered using a high-pass frequency filter at 0.1 Hz, a low-pass frequency filter at 40 Hz, and a 60-Hz notch filter. Ocular correction was accomplished via detection and rectification of blinks and horizontal eye movements using the VEOG and HEOG channels (Gratton et al., 1983). Semi-automatic artifact rejection was then used to remove channels by segment with the following criteria for automatic artifact rejection: voltage steps exceeding 30 µV, changes within a given segment greater than 150 µV, and activity under.5 µV persistent for 100 ms or more. Additional artifacts were identified by visual inspection and removed. All artifacting was completed using Brain Vision Analyzer (BVA; Brain Products GmbH). Spectral analysis was conducted to generate delta-beta coupling and frontal asymmetry metrics. Artifact-free data were submitted to Fast-Fourier Transformation (FFT) with a 50 % Hamming window overlap. Spectral power density (µV^2^/Hz) was assessed for each frequency band and exported as mean power separately for delta (1–4 Hz), alpha (8–13 Hz), and beta (13–25 Hz) frequency bands.

Delta and beta power were extracted from a cluster of frontal sites (F3, Fz, F4). A natural log (ln) transformation was applied separately to each frequency band across participants to normalize the distributions and reduce skewness, following several other studies of delta-beta coupling (Morillas-Romero et al., 2015, Phelps et al., 2016, Poole et al., 2020, Vanpeer, 2008). The resulting delta and beta ranges were as follows: delta (1.03 – 4.57; *M* = 2.42, *SD* = 0.73); beta (0.16 – 1.33; *M* = 0.50, *SD* = 0.26), which is consistent with ranges found in other samples with similar processing steps (e.g., Poole et al., 2020). Moreover, second-by-second delta and beta power demonstrated excellent internal consistency across the task (Cronbach’s alpha = .991 for delta and .998 for beta).

Following prior studies (Myruski et al., 2022; 2024), delta-beta coupling was calculated as the absolute value of the residual score derived from a linear regression with delta predicting beta. This approach allowed us to capture the coherence between delta and beta power for each individual, rather than deriving a group-level estimate of delta-beta coupling as is traditionally done, sometimes with arbitrary distinctions such as a median or mean split (e.g., Brooker et al., 2016; Phelps et al., 2016; Putman, 2011). We specifically computed the residual for delta predicting beta because we wanted to capture the degree to which higher-order oscillations (i.e., beta) showed activation over and above subcortical oscillations associated with limbic activity (i.e., delta). This is consistent with the conceptualization of high delta-beta coupling as reflecting top-down overcontrol (Myruski et al., 2024, Phelps et al., 2016, Schutter and Knyazev, 2012). To aid interpretation, scores were reversed such that higher values reflect stronger delta-beta coupling.

Frontal asymmetry was calculated at frontal scalp sites by subtracting the alpha band power values from a cluster of electrodes in the left hemisphere (Fp1, FC1, FC5, F3, F7) from a homologous cluster in the right hemisphere (Fp2, FC2, FC6, F4, F8). These clusters were selected based on visual inspection of the scalp distribution of alpha power, which was maximal across these electrode sites (as indicated by the lowest levels of alpha activation; see Figure S1). Therefore, more negative scores, or in other words greater left alpha power than right alpha power, indicate heightened neural activation on the right hemisphere.

## 3 Results

### 3.1 Analytic approach

#### 3.1.1 Handling missing data

SPSS Version 27 was used to evaluate missingness and conduct multiple imputation. Overall, the missingness percentage was 3.54 % across all variables. The majority of participants (*n* = 91, 93.81 %) had complete data, and 6 (6.17 %) had missing values, all of which did not complete the SAS-A questionnaire. Little’s test was non-significant [χ^2^(3) = 0.83, *p* = .84], indicating that the pattern of missingness may be MCAR (i.e., missing completely at random), lending support for the use of multiple imputation to account for missing values. Multiple imputation was conducted using 20 imputations following evidence (Graham et al., 2007) that this number yields similar results as other approaches to handling missing data (e.g., full information maximum likelihood). Analyses reported below used pooled data, and we also confirmed that models run with non-imputed data yielded the same patterns and levels of significance.

Descriptive statistics and correlations among study variables are presented in Table 1.

**Table 1:** **Descriptive Statistics and Correlations**.

*Variable*	Descriptive Statistics			Correlations *r (p)*						
	*M (SD)*	*Min*	*Max*	*1*	*2*	*3*	*4*	*5*	*6*	*7*
1. Delta-Beta Coupling	−0.73 (0.55)	−2.28	−0.01	-	−.15(.143)	.08(.420)	**.29(.005)**	**.26(.012)**	**.30(.003)**	**.31(.002)**
2. Frontal Asymmetry	0.01 (0.42)	−1.20	1.45		-	.02(.876)	−.14(.187)	−.11(.301)	−.09(.375)	−.06(.589)
3. Behavioral Inhibition	112.82 (35.28)	33.00	177.00			-	**.40(<.001)**	**.23(.021)**	**.52(<.001)**	**.22(.033)**
4. Total Social Anxiety Symptoms	54.74 (12.84)	26.00	87.00				-	**.91(<.001)**	**.83(<.001)**	**.59(<.001)**
5. Fear of Negative Evaluation	22.05 (8.40)	8.00	40.00					-	**.69(<.001)**	**.61(<.001)**
6. Avoidance/Distress (Novelty)	19.67 (6.74)	6.00	40.00						-	**.74(<.001)**
7. Avoidance/Distress (General)	11.55 (6.50)	4.00	38.00							-

#### 3.1.2 Main analyses

Moderation analyses were conducted using SPSS PROCESS Macro v4.2. We first examined whether age, sex, or pubertal development should be included as covariates by testing their associations with our predictor variables, which were all nonsignificant (*p*s > .08). Therefore, age,sex, and pubertal development were not included as covariates to maximize the parsimony and interpretability of subsequent models. DBC and frontal asymmetry were tested individually as moderators of the BI-SA association using PROCESS Model 1. Separate models (4 models each for DBC and frontal asymmetry, 8 models total) were conducted for each dependent variable capturing social anxiety symptoms (i.e., total symptoms, fear of negative evaluation, avoidance and distress to social novelty, avoidance and distress to social situations generally). Due to the large number of analyses in the study, we utilized the Benjamini-Hochberg procedure to control for a 5 % false discovery rate (Benjamini and Hochberg, 1995). *P*-values greater than 0.02 were labeled as not significant. All predictors were mean-centered prior to analysis. Full model statistics are presented in Table 2, Table 3.

**Table 2:** Delta-Beta Coupling Moderation Models.

**2a. Outcome: Total Social Anxiety Symptoms**						

*Model Statistics*	*R*	*R*^*2*^	*MSE*	*F*	*df*	*p*

	.47	.22	131.97	8.99	3, 93	**< .001**

*Predictors*	*Coeff*	*SE*	*t*	*p*	*LLCI*	*ULCI*

Constant	54.79	1.17	46.82	**< .001**	52.47	57.12
BI	0.14	0.03	4.03	**< .001**	0.07	0.20
DBC	5.98	2.13	2.81	**.006**	1.76	10.20
BI*DBC	−0.13	0.06	−0.54	.593	−0.15	0.09

**2b. Outcome: Fear of Negative Evaluation**						

*Model Statistics*	*R*	*R*^*2*^	*MSE*	*F*	*df*	*p*

	.34	.12	64.50	4.03	3, 93	**.010**

*Predictors*	*Coeff*	*SE*	*t*	*p*	*LLCI*	*ULCI*

Constant	22.09	0.82	27.00	**< .001**	20.47	23.72
BI	0.05	0.02	2.10	.038	0.00	0.10
DBC	3.68	1.49	2.47	**.015**	0.73	6.63
BI*DBC	−0.03	0.04	−0.69	.493	−0.11	0.05

**2c. Outcome: Avoidance and Distress - Novelty**						

*Model Statistics*	*R*	*R*^*2*^	*MSE*	*F*	*df*	*p*

	.58	.33	31.40	15.36	3, 93	**< .001**

*Predictors*	*Coeff*	*SE*	*t*	*p*	*LLCI*	*ULCI*

Constant	19.68	0.57	34.48	**< .001**	18.55	20.82
BI	0.09	0.02	5.73	**< .001**	0.06	0.13
DBC	3.15	1.04	3.04	**.003**	1.09	5.21
BI*DBC	−0.01	0.03	−0.35	.729	−0.07	0.05

**2d. Outcome: Avoidance and Distress - General**						

*Model Statistics*	*R*	*R*^*2*^	*MSE*	*F*	*df*	*p*

	.37	.14	37.57	4.98	3, 93	**.003**

*Predictors*	*Coeff*	*SE*	*t*	*p*	*LLCI*	*ULCI*

Constant	11.59	0.62	18.57	**< .001**	10.35	12.83
BI	0.03	0.02	1.89	.062	0.00	0.07
DBC	3.51	1.14	3.09	**.003**	1.26	5.76
BI*DBC	−0.03	0.03	−0.83	.411	−0.09	0.04

Note. BI = Behavioral Inhibition; DBC = Delta-Beta Coupling. Bold values indicate a significant effect (*p* < .02).

**Table 3:** Frontal Asymmetry Moderation Models.

**3a. Outcome: Total Social Anxiety Symptoms**						

*Model Statistics*	*R*	*R*^*2*^	*MSE*	*F*	*df*	*p*

	.42	.18	139.83	6.74	3, 93	**< .001**

*Predictors*	*Coeff*	*SE*	*t*	*p*	*LLCI*	*ULCI*

Constant	54.75	1.20	45.59	**< .001**	52.36	57.13
BI	0.14	0.03	4.20	**< .001**	0.08	0.21
Asym	−4.39	2.88	−1.52	.131	−10.12	1.33
BI*Asym	−0.02	0.07	−0.25	.800	−0.15	0.12

**3b. Outcome: Fear of Negative Evaluation**						

*Model Statistics*	*R*	*R*^*2*^	*MSE*	*F*	*df*	*p*
	.28	.08	67.37	2.55	3, 93	.061

*Predictors*	*Coeff*	*SE*	*t*	*p*	*LLCI*	*ULCI*

Constant	22.06	0.83	26.46	**< .001**	20.40	23.71
BI	0.05	0.02	2.24	.028	0.01	0.10
Asym	−2.40	2.00	−1.20	.233	−6.38	1.57
BI*Asym	−0.05	0.05	−0.97	.336	−0.14	0.05

**3c. Outcome: Avoidance and Distress - Novelty**						

*Model Statistics*	*R*	*R*^*2*^	*MSE*	*F*	*df*	*p*

	.53	.28	33.91	11.93	3, 93	**< .001**

*Predictors*	*Coeff*	*SE*	*t*	*p*	*LLCI*	*ULCI*

Constant	19.67	0.59	33.27	**< .001**	18.50	20.85
BI	0.10	0.02	5.75	**< .001**	0.06	0.13
Asym	−1.69	1.42	−1.19	.237	−4.51	1.13
BI*Asym	−0.02	0.03	−0.64	.522	−0.09	0.05

**3d. Outcome: Avoidance and Distress - General**						

*Model Statistics*	*R*	*R*^*2*^	*MSE*	*F*	*df*	*p*

	.31	.09	39.51	3.22	3, 93	**.026**

*Predictors*	*Coeff*	*SE*	*t*	*p*	*LLCI*	*ULCI*

Constant	11.57	0.64	18.13	**< .001**	10.30	12.84
BI	0.04	0.02	1.96	.054	0.00	0.07
Asym	−1.27	1.53	−0.83	.411	−4.31	1.78
BI*Asym	−0.08	0.04	−2.12	.037	−0.15	0.00

Note. BI = Behavioral Inhibition; Asym = Frontal Asymmetry. Bold values indicate a significant effect (*p* < .02).

### 3.2 Delta-beta coupling

DBC significantly directly predicted all four social anxiety symptom scales, with higher coupling being associated with greater total symptoms (b = 5.78, *SE* = 2.13, *p* = .006), fear of negative evaluation (b = 3.68, *SE* = 1.49, *p* = .015), avoidance and distress to social novelty (b = 3.15, *SE* = 1.04, *p* = .003), and avoidance and distress to social situations generally (b = 3.51, *SE* = 1.14, *p* = .003). Higher BI was significantly associated with greater total symptoms (b = 0.14, *SE* = 0.03, *p* < .001) and greater avoidance and distress to social novelty (b = 0.09, *SE* = 0.02, *p* < .001). BI was not significantly associated with fear of negative evaluation (b = 0.05, *SE* = 0.02, *p* = .038) or avoidance and distress to social situations generally (b = 0.04, *SE* = 0.02, *p* = .062), nor did DBC significantly interact with BI to predict social anxiety symptoms for any scale (*p’s* > .411).

### 3.3 Frontal asymmetry

In contrast, frontal asymmetry did not significantly directly predict social anxiety symptoms (*p’s* > .131). Similar to the DBC models, the models with frontal asymmetry showed that greater BI significantly predicted greater total symptoms (b = 0.14, *SE* = 0.03, *p* = .001) and greater avoidance and distress to social novelty (b = 0.10, *SE* = 0.02, *p* < .001), but BI was not significantly associated with fear of negative evaluation (b = 0.05, *SE* = 0.02, *p* = .028) or avoidance and distress to social situations generally (b = 0.04, *SE* = 0.02, *p* = .054).

**Fig. 1:**
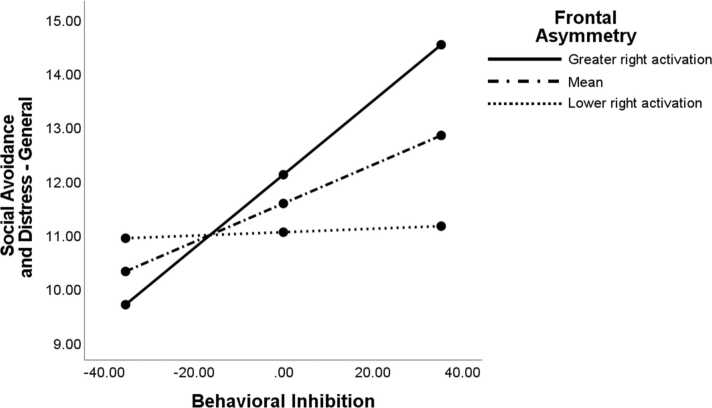
**2-way interaction between BI and frontal alpha asymmetry predicting social anxiety symptoms.***Note*: The relation between BI and general social avoidance and distress was significant only at high (+1 SD) levels of relative right activation (*β* =.07, SE =.02, *p* = .003), but not at the mean (*β* =.04, SE =.01, *p* = .054) or at low (-1 SD) levels of relative right activation (*β* =.00, SE =.03, *p* = .90).

**Fig. 2:**
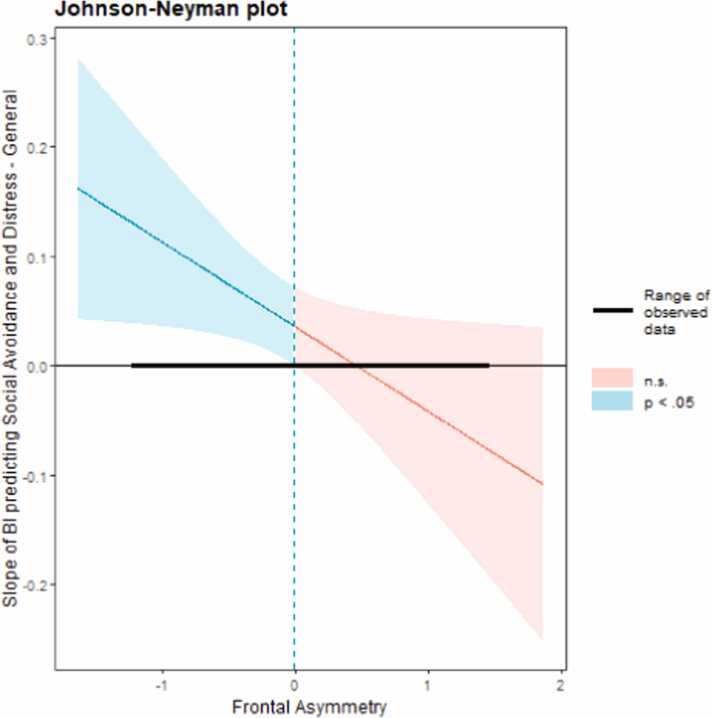
Johnson-Neyman region of significance for the association between BI and social anxiety at varying levels of frontal asymmetry.

The interaction between frontal asymmetry and BI predicting avoidance and distress to social situations generally was statistically significant but did not survive our correction for multiple comparisons [BI*Asym: b = -0.08, SE = 0.04, ∆*R*^2^ = .04, *F*(1, 93) = 4.50, *p* = .037]. Consequently, this effect should be interpreted with caution, but we did proceed to probe this interaction given our a priori theoretical predictions (Fig. 1). Consistent with our hypothesis, the Johnson-Neyman region of significance analysis revealed that the frontal asymmetry moderator became significant at values of −.007 and below, indicating that greater relative right activation predicted a stronger BI-SA link (Fig. 2).

### 3.4 Exploratory analyses

Given epidemiological evidence of sex differences in the emergence of social anxiety during early adolescence (Asher et al., 2017) and developmental differences in brain circuitry underlying emotion regulation (Goddings et al., 2014, Mills et al., 2014), we computed exploratory analyses examining the moderating effects of sex, age, and pubertal development on the reported findings (see Supplemental Materials for the full statistics). There was a significant three-way interaction between frontal alpha asymmetry, age, and sex predicting avoidance and distress to social situations generally [B = 6.91, SE = 3.38, ∆*R*^2^ = .03, *F*(1, 89) = 4.19, *p* = .044], such that greater relative right activation significantly predicted social anxiety only for early adolescent girls (at age = 13.40 years; simple slope = −6.99, SE = 2.89, *p* = .02). Additionally, there was a significant three-way interaction between BI, frontal alpha asymmetry, and age predicting total social anxiety symptoms [B = 0.16, SE = 0.08, ∆*R*^2^ = .03, *F*(1, 89) = 4.05, *p* = .047], such that the BI by asymmetry interaction was strongest for early adolescents (age = 13.40 years). Of note, neither of these exploratory findings survived our correction for multiple comparisons. No other significant interactions with sex, age, or pubertal development emerged (*p*s > .09).

## 4 Discussion

The overarching goal of the current study was to examine the role of resting-state oscillatory patterns on the association between adolescent BI and social anxiety symptoms. Results indicated that stronger DBC was directly associated with higher social anxiety symptoms, but not BI, and did not moderate the association between BI and social anxiety. In contrast, frontal alpha asymmetry was not directly associated with either BI or social anxiety but interacted with BI to predict avoidance and distress to social situations, such that greater relative right activation predicted a stronger BI-social anxiety link. However, this interaction effect did not survive correction for multiple comparisons. These findings suggest that high DBC may mark a general vulnerability for social anxiety symptoms, perhaps indexing difficulties with emotion regulation. Conversely, frontal alpha asymmetry may potentiate the risk for social anxiety symptoms specifically among BI youth, potentially by supporting avoidance behaviors, although replication of this effect in other samples is warranted.

We did not find a significant association between delta-beta coupling and adolescent BI. This is inconsistent with prior work in early childhood samples, which has found that BI is associated with stronger delta-beta coupling (Phelps et al., 2016, Poole et al., 2020). One possibility for these disparate findings is that there are developmental changes in the association between BI and delta-beta coupling, such that this relation is observed earlier in development (Anaya et al., 2021a, Poole et al., 2020) but not by adolescence, when DBC may begin to better characterize anxiety symptoms rather than temperamental vulnerability. Consistent with this interpretation, other work has shown that greater delta-beta coupling is associated with higher social anxiety symptoms, but not BI, in early adolescence (Anaya et al., 2021b). This could be because BI youth exhibit patterns of delta-beta coupling usually not observed until adolescence, at which point normative developmental increases in cortical regulation may normalize delta-beta coupling patterns across youth at high and low temperamental risk. By adolescence, relatively high DBC may instead reflect a pattern of cortical overcontrol that underlies the more severe functional impairment or distress characteristic of anxiety. Longitudinal work is needed to directly test these hypotheses.

Even after accounting for BI, delta-beta coupling directly predicted social anxiety symptoms but did not interact with BI to predict social anxiety symptoms. These findings suggest that, at least in adolescence, delta-beta coupling may be a marker of anxiety rather than temperamental vulnerability. Delta-beta coupling indexes coherence between cortical and subcortical brain regions, relevant to higher-order processing and limbic functioning, respectively (Schutter and Knyazev, 2012). Therefore, the present findings are consistent with other studies showing that anxiety is associated with exaggerated prefrontal activation and greater connectivity between the prefrontal cortex and amygdala during threat processing (Monk et al., 2006, Monk et al., 2008, Telzer et al., 2008).

Higher delta-beta coupling at rest may reflect an underlying disposition to engage cortical processes in response to limbic activation, which may be more reflective of adolescent anxiety phenotypes than BI, which is characterized by hypervigilance and excessive reactivity of fear-related circuitry (Blackford et al., 2011, Kagan and Snidman, 1991, Schwartz et al., 2003). Indeed, our findings suggest that this neural pattern is a general marker of social anxiety symptoms that predicts risk over and above temperamental vulnerabilities, rather than a moderator of the BI-anxiety link. Behaviorally, stronger delta-beta coupling may manifest as a proclivity for more controlled social interactions, as has been shown in our own work demonstrating that delta-beta coupling mediates the relation between preference for digital media use and social anxiety symptoms (Myruski et al., 2024).

We did not find evidence that EEG asymmetry is directly related to BI or social anxiety symptoms during adolescence. However, post hoc analyses indicated that greater relative right frontal activity increased the association between adolescent BI and social avoidance and distress symptoms, which was consistent with our hypothesis. These findings are consistent with conceptual models proposing that frontal asymmetry is better represented as a moderator or mediator of outcomes rather than a direct predictor (Reznik and Allen, 2018). Further, consistent with prior work (Buzzell et al., 2017, Harrewijn et al., 2019), these results suggest that frontal asymmetry reflects a vulnerability to social anxiety development for temperamentally fearful adolescents in particular. Interestingly, our exploratory analyses showed that frontal asymmetry was particularly predictive of higher social anxiety symptoms for younger adolescents, which could be because the maturation of cortico-limbic connections evident later buffers this heightened sensitivity to threat indexed by greater relative right activation (Heffer and Willoughby, 2020, Mills et al., 2014).

Given the absence of a direct association with social anxiety symptoms, frontal alpha asymmetry may better reflect a behavioral or temperamental tendency to avoid rather than directly characterizing functional impairment associated with clinical levels of anxiety. This specificity for general avoidance and distress aligns with decades of work finding that greater right frontal asymmetry reflects a withdrawal and avoidance bias (Buss et al., 2003, Davidson, 1998, McManis et al., 2002). It is possible that other neural markers, such as indicators of error monitoring or hypervigilance toward social threat, can differentiate other components of social anxiety such as fear of negative evaluation, which is an important direction for future work.

In the extant literature, the magnitude of the association as well as shared phenotypic measurement of BI and social anxiety symptoms (i.e., social avoidance and fear of evaluation) has raised questions about whether BI is simply a prodromal form of social anxiety (Pérez-Edgar and Guyer, 2014). We found that the resting-state measures were differentially associated with BI and social anxiety symptoms. Specifically, asymmetry was not directly associated with BI or social anxiety symptoms, and delta-beta coupling was directly associated with social anxiety symptoms, but not BI. Moreover, only asymmetry was found to moderate the BI-social anxiety link, suggesting that asymmetry may be a stronger marker of temperamental vulnerability for social anxiety. The pattern of findings in the current study is consistent with other work that demonstrates that BI, asymmetry, and delta-beta coupling are unique markers of predisposing vulnerabilities to the development of anxiety symptoms (Klein and Mumper, 2018, Pérez-Edgar and Fox, 2018) and are not interchangeable constructs. Thus, the current study adds to the evidence that BI and social anxiety disorder are separate phenotypes. While there are phenotypic similarities in some associated behaviors, social anxiety disorder uniquely includes functional impairment which is a necessary component for diagnosis.

A few limitations were inherent in the present study and should guide future work. First, we used cross-sectional data to address our research questions and consequently cannot make strong claims about trajectories of social anxiety across adolescence or the temporal associations between our study variables. Relatedly, it is possible that our BI measure was confounded by the onset of social anxiety symptoms. At the same time, assessing our study variables contemporaneously allowed us to disentangle neural correlates of BI and social anxiety while controlling for potential age-related differences in these neural measures. However, future work could examine at what point in development these associations begin to emerge in order to facilitate early detection of risk. Second, BI was assessed via parent report, whereas social anxiety symptoms were self-reported by adolescents. These informants were selected to be consistent with prior studies of adolescent temperament and social anxiety (e.g., Murillo et al., 2024) and to reflect the emphasis in BI on external behavior, and in social anxiety on internal feelings and cognitions. It is possible that BI and social anxiety were differentially associated with the neural measures due to cross-informant variance. Third, we used average alpha asymmetry and DBC scores across the baseline task because we were interested in how between-person differences in these neural measures relate to BI and social anxiety. However, other work suggests that assessing these metrics across different time scales (e.g., second-to-second/within-person, developmental time scales), may reveal unique associations with BI and social anxiety (Anaya et al., 2021b, Perone and Vaughan, 2024). Finally, our sample size was smaller than planned due to the COVID-19 pandemic. While a sensitivity power analysis indicated that we were sufficiently powered for the current analyses, replication in larger samples or in samples with differing demographic characteristics is warranted.

## 5 Conclusions

The present study advances extant literature by clarifying the role of frontal EEG asymmetry and delta-beta coupling in the link between BI and various presentations of social anxiety symptoms during adolescence. Delta-beta coupling was directly associated with social anxiety symptoms, but not BI, and did not moderate the BI-social anxiety link. In contrast, greater relative right frontal alpha asymmetry strengthened the association between BI and social anxiety symptoms, specifically generalized avoidance and distress in social situations, but was not directly associated with BI or social anxiety symptoms. These findings suggest unique roles of delta-beta coupling and frontal alpha asymmetry in conferring risk for social anxiety symptoms during adolescence. Specifically, high delta-beta coupling may mark a general vulnerability for social anxiety symptoms, whereas frontal alpha asymmetry may compound the risk for social anxiety symptoms and functional impairment specifically among BI youth, potentially by supporting avoidance behaviors.

## Declaration of Competing Interest

The authors declare that they have no known competing financial interests or personal relationships that could have appeared to influence the work reported in this paper.

## Data Availability

Data will be shared upon completion of the longitudinal study through NIH-NDA.
