# Supplementary material for: Disentangling the role of different resting-state neural markers of adolescent behavioral inhibition and social anxiety
Source: Dev Cogn Neurosci. 2025 Apr 24;73:101560. doi: 10.1016/j.dcn.2025.101560 (PMC12063126; doi:10.1016/j.dcn.2025.101560)
Supplement: Supplementary file 1 — Supplementary material [file mmc1.docx]

**Table S1**

**Moderating Effects of Age, Sex, and Pubertal Development on Frontal Asymmetry Models**

| **Outcome: Total Social Anxiety Symptoms** | |  | |  |  | |  | |  | |  | |  |  |
| --- | --- | --- | --- | --- | --- | --- | --- | --- | --- | --- | --- | --- | --- | --- |
|  | *Predictor* | | *Coeff* | | | *SE* | | *t* | | *p* | | *LLCI* | | *ULCI* |
| Step 1 | BI | | 0.13 | | | 0.03 | | 4.12 | | **<.001** | | 0.07 | | 0.20 |
|  | Asym | | -6.99 | | | 7.35 | | -0.95 | | .34 | | -21.60 | | 7.61 |
|  | Age | | 4.51 | | | 1.30 | | 3.46 | | **.001** | | 1.92 | | 7.09 |
|  | Sex | | 9.12 | | | 2.74 | | 3.33 | | **.001** | | 3.67 | | 14.56 |
|  | PubDev | | -2.73 | | | 2.18 | | -1.25 | | .21 | | -7.05 | | 1.60 |
| Step 2 | BI*Asym | | 0.00 | | | 0.06 | | 0.02 | | .98 | | -.12 | | .13 |
|  | Asym*Age | | -1.48 | | | 3.34 | | -0.44 | | .66 | | -8.12 | | 5.16 |
|  | Asym*Sex | | -6.19 | | | 5.29 | | -1.17 | | .25 | | -16.71 | | 4.32 |
|  | Asym*PubDev | | -3.71 | | | 4.14 | | -0.90 | | .37 | | -11.93 | | 4.52 |
| Step 3 | Asym*Sex*Age | | -1.34 | | | 7.43 | | -0.18 | | .86 | | -16.10 | | 13.42 |
|  | Asym*Sex*PubDev | | -0.77 | | | 14.39 | | -0.05 | | .96 | | -29.37 | | 27.83 |
|  | BI*Asym*Age | | 0.16 | | | 0.08 | | 2.01 | | **.047** | | .002 | | .31 |
|  | BI*Asym*PubDev | | 0.13 | | | 0.13 | | 0.96 | | .34 | | -0.13 | | 0.39 |
|  | BI*Asym*Sex | | -0.02 | | | 0.14 | | -0.15 | | .88 | | -0.30 | | 0.26 |
| **Outcome: Fear of Negative Evaluation** | |  | |  |  | |  | |  | |  | |  |  |
|  | *Predictor* | | *Coeff* | | | *SE* | | *t* | | *p* | | *LLCI* | | *ULCI* |
| Step 1 | BI | | 0.05 | | | 0.02 | | 2.14 | | **.035** | | .003 | | .09 |
|  | Asym | | -0.78 | | | 4.99 | | -0.16 | | .88 | | -10.69 | | 9.13 |
|  | Age | | 3.72 | | | 0.88 | | 4.21 | | **<.001** | | 1.96 | | 5.47 |
|  | Sex | | 5.42 | | | 1.86 | | 2.91 | | **.005** | | 1.72 | | 9.11 |
|  | PubDev | | -1.44 | | | 1.48 | | -0.97 | | .33 | | -4.38 | | 1.50 |
| Step 2 | BI*Asym | | -0.03 | | | 0.04 | | -0.71 | | .48 | | -0.12 | | 0.05 |
|  | Asym*Age | | -0.89 | | | 2.27 | | -0.39 | | .70 | | -5.41 | | 3.63 |
|  | Asym*Sex | | -5.82 | | | 3.58 | | -1.63 | | .11 | | -12.92 | | 1.29 |
|  | Asym*PubDev | | -2.39 | | | 2.82 | | -0.85 | | .40 | | -7.99 | | 3.21 |
| Step 3 | Asym*Sex*Age | | .89 | | | 5.02 | | 0.18 | | .86 | | -9.10 | | 10.87 |
|  | Asym*Sex*PubDev | | -5.48 | | | 9.69 | | -0.57 | | .57 | | -24.74 | | 13.78 |
|  | BI*Asym*Age | | 0.07 | | | 0.05 | | 1.24 | | .22 | | -0.04 | | 0.17 |
|  | BI*Asym*PubDev | | 0.03 | | | 0.09 | | 0.39 | | .70 | | -0.14 | | 0.21 |
|  | BI*Asym*Sex | | 0.01 | | | 0.10 | | 0.06 | | .96 | | -0.19 | | 0.20 |
| **Outcome: Avoidance and Distress - Novelty** | |  | |  |  | |  | |  | |  | |  |  |
|  | *Predictor* | | *Coeff* | | | *SE* | | *t* | | *p* | | *LLCI* | | *ULCI* |
| Step 1 | BI | | 0.10 | | | 0.02 | | 6.58 | | **<.001** | | 0.07 | | 0.13 |
|  | Asym | | -1.96 | | | 3.48 | | -0.56 | | .58 | | -8.88 | | 4.96 |
|  | Age | | 3.28 | | | 0.62 | | 5.33 | | **<.001** | | 2.06 | | 4.51 |
|  | Sex | | 1.28 | | | 1.30 | | 0.98 | | .33 | | -1.30 | | 3.86 |
|  | PubDev | | -1.07 | | | 1.03 | | -1.03 | | .30 | | -3.12 | | 0.98 |
| Step 2 | BI*Asym | | -0.01 | | | 0.03 | | -0.23 | | .82 | | -0.07 | | 0.05 |
|  | Asym*Age | | -1.85 | | | 1.57 | | -1.18 | | .24 | | -4.98 | | 1.27 |
|  | Asym*Sex | | 0.06 | | | 2.52 | | 0.02 | | .98 | | -4.96 | | 5.08 |
|  | Asym*PubDev | | -1.47 | | | 1.96 | | -0.75 | | .45 | | -5.38 | | 2.43 |
| Step 3 | Asym*Sex*Age | | 1.16 | | | 3.49 | | 0.33 | | .74 | | -5.79 | | 8.11 |
|  | Asym*Sex*PubDev | | -2.47 | | | 6.78 | | -0.36 | | .72 | | -15.95 | | 11.01 |
|  | BI*Asym*Age | | 0.06 | | | 0.03 | | 1.67 | | 0.10 | | -0.01 | | 0.13 |
|  | BI*Asym*PubDev | | 0.11 | | | 0.06 | | 1.94 | | .06 | | -0.003 | | 0.22 |
|  | BI*Asym*Sex | | 0.07 | | | 0.07 | | 1.03 | | .31 | | -0.07 | | 0.22 |
| **Outcome: Avoidance and Distress - General** | |  | |  |  | |  | |  | |  | |  |  |
|  | *Predictor* | | *Coeff* | | | *SE* | | *t* | | *p* | | *LLCI* | | *ULCI* |
| Step 1 | BI | | 0.04 | | | 0.01 | | 3.11 | | **.003** | | 0.02 | | 0.07 |
|  | Asym | | 4.01 | | | 3.29 | | 1.22 | | .23 | | -2.53 | | 10.55 |
|  | Age | | 4.56 | | | 0.58 | | 7.82 | | **<.001** | | 3.40 | | 5.71 |
|  | Sex | | -0.11 | | | 1.23 | | -0.09 | | .93 | | -2.55 | | 2.33 |
|  | PubDev | | -1.51 | | | 0.98 | | -1.55 | | .13 | | -3.45 | | 0.43 |
| Step 2 | BI*Asym | | -0.06 | | | 0.03 | | -2.03 | | **.046** | | -0.11 | | -0.001 |
|  | Asym*Age | | 0.23 | | | 1.53 | | 0.15 | | .88 | | -2.82 | | 3.27 |
|  | Asym*Sex | | -1.15 | | | 2.44 | | -0.47 | | .64 | | -6.00 | | 3.70 |
|  | Asym*PubDev | | -1.00 | | | 1.90 | | -0.53 | | .60 | | -4.78 | | 2.78 |
| Step 3 | Asym*Sex*Age | | 6.91 | | | 3.38 | | 2.05 | | **.044** | | 0.20 | | 13.63 |
|  | Asym*Sex*PubDev | | -1.43 | | | 6.54 | | -0.22 | | .83 | | -14.43 | | 11.56 |
|  | BI*Asym*Age | | 0.00 | | | 0.04 | | 0.14 | | .89 | | -0.07 | | 0.07 |
|  | BI*Asym*PubDev | | 0.00 | | | 0.06 | | 0.08 | | .94 | | -0.11 | | 0.11 |
|  | BI*Asym*Sex | | 0.03 | | | 0.08 | | 0.39 | | .70 | | -0.13 | | 0.19 |

*Note*: Interaction terms were tested in individual models to avoid overcontrolling and maximize power; BI = behavioral inhibition; Asym = frontal alpha asymmetry; PubDev = pubertal development; Biological sex was coded as 1 = male, 2 = female.

**Table S2**

**Moderating Effects of Age, Sex, and Pubertal Development on Delta-Beta Coupling Models**

| **Outcome: Total Social Anxiety Symptoms** | |  |  | |  | |  | |  | |  | |  |  |
| --- | --- | --- | --- | --- | --- | --- | --- | --- | --- | --- | --- | --- | --- | --- |
|  | *Predictor* | | | *Coeff* | | *SE* | | *t* | | *p* | | *LLCI* | | *ULCI* |
| Step 1 | BI | | | 0.13 | | 0.05 | | 2.45 | | **.016** | | 0.02 | | 0.24 |
|  | DBC | | | 4.50 | | 6.77 | | 0.66 | | .51 | | -8.96 | | 17.96 |
|  | Age | | | 3.63 | | 1.29 | | 2.82 | | **.006** | | 1.08 | | 6.19 |
|  | Sex | | | 8.67 | | 2.74 | | 3.16 | | **.002** | | 3.22 | | 14.12 |
|  | PubDev | | | -2.42 | | 2.17 | | -1.11 | | .27 | | -6.74 | | 1.90 |
| Step 2 | BI*DBC | | | 0.01 | | 0.06 | | 0.17 | | .86 | | -0.10 | | 0.12 |
|  | DBC*Age | | | -1.30 | | 2.32 | | -.56 | | .58 | | -5.91 | | 3.31 |
|  | DBC*Sex | | | -0.88 | | 4.04 | | -.22 | | .83 | | -8.90 | | 7.14 |
|  | DBC*PubDev | | | -1.51 | | 2.62 | | -.58 | | .56 | | -6.72 | | 3.69 |
| Step 3 | DBC*Sex*Age | | | -0.34 | | 4.90 | | -.07 | | .95 | | -10.08 | | 9.41 |
|  | DBC*Sex*PubDev | | | -0.04 | | 6.71 | | -.01 | | .99 | | -13.37 | | 13.29 |
|  | BI*DBC*Age | | | 0.04 | | 0.07 | | 0.61 | | .54 | | -0.10 | | 0.19 |
|  | BI*DBC*PubDev | | | -0.06 | | 0.08 | | -0.73 | | .47 | | -0.22 | | 0.10 |
|  | BI*DBC*Sex | | | -0.10 | | 0.12 | | -0.81 | | .42 | | -0.34 | | 0.14 |
| **Outcome: Fear of Negative Evaluation** | |  |  | |  | |  | |  | |  | |  |  |
|  | *Predictor* | | | *Coeff* | | *SE* | | *t* | | *p* | | *LLCI* | | *ULCI* |
| Step 1 | BI | | | 0.04 | | 0.04 | | 1.18 | | .24 | | -.03 | | .11 |
|  | DBC | | | 3.24 | | 4.63 | | 0.70 | | .49 | | -5.96 | | 12.43 |
|  | Age | | | 3.23 | | 0.88 | | 3.68 | | **<.001** | | 1.49 | | 4.98 |
|  | Sex | | | 5.11 | | 1.88 | | 2.72 | | **.008** | | 1.34 | | 8.83 |
|  | PubDev | | | -1.23 | | 1.49 | | -0.83 | | .41 | | -4.18 | | 1.72 |
| Step 2 | BI*DBC | | | 0.00 | | 0.04 | | -0.01 | | .99 | | -0.08 | | 0.08 |
|  | DBC*Age | | | -0.90 | | 1.59 | | -0.57 | | .57 | | -4.05 | | 2.24 |
|  | DBC*Sex | | | -0.80 | | 2.76 | | -0.29 | | .77 | | -6.28 | | 4.68 |
|  | DBC*PubDev | | | -1.80 | | 1.78 | | -1.01 | | .32 | | -5.34 | | 1.75 |
| Step 3 | DBC*Sex*Age | | | 1.59 | | 3.35 | | 0.48 | | .64 | | -5.07 | | 8.25 |
|  | DBC*Sex*PubDev | | | 0.37 | | 4.56 | | 0.08 | | .93 | | -8.69 | | 9.44 |
|  | BI*DBC*Age | | | 0.04 | | 0.05 | | 0.89 | | .37 | | -0.05 | | 0.14 |
|  | BI*DBC*PubDev | | | -0.03 | | 0.06 | | -0.44 | | .66 | | -0.14 | | 0.09 |
|  | BI*DBC*Sex | | | -0.05 | | 0.08 | | -0.54 | | .59 | | -0.21 | | 0.12 |
| **Outcome: Avoidance and Distress - Novelty** | |  |  | |  | |  | |  | |  | |  |  |
|  | *Predictor* | | | *Coeff* | | *SE* | | *t* | | *p* | | *LLCI* | | *ULCI* |
| Step 1 | BI | | | 0.10 | | 0.03 | | 3.94 | | **<.001** | | 0.05 | | 0.15 |
|  | DBC | | | 2.14 | | 3.18 | | 0.67 | | .50 | | -4.18 | | 8.46 |
|  | Age | | | 2.93 | | .60 | | 4.84 | | **<.001** | | 1.73 | | 4.13 |
|  | Sex | | | 1.14 | | 1.29 | | 0.88 | | .38 | | -1.43 | | 3.70 |
|  | PubDev | | | -0.96 | | 1.02 | | -0.94 | | .35 | | -2.99 | | 1.06 |
| Step 2 | BI*DBC | | | 0.00 | | 0.03 | | 0.15 | | .88 | | -0.05 | | 0.06 |
|  | DBC*Age | | | -0.33 | | 1.09 | | -0.31 | | .76 | | -2.50 | | 1.83 |
|  | DBC*Sex | | | -1.93 | | 1.89 | | -1.02 | | .31 | | -5.67 | | 1.82 |
|  | DBC*PubDev | | | -1.11 | | 1.23 | | -0.91 | | .37 | | -3.55 | | 1.32 |
| Step 3 | DBC*Sex*Age | | | 0.12 | | 2.27 | | 0.05 | | .96 | | -4.39 | | 4.62 |
|  | DBC*Sex*PubDev | | | -0.06 | | 3.09 | | -0.02 | | .98 | | -6.20 | | 6.08 |
|  | BI*DBC*Age | | | 0.01 | | 0.03 | | 0.44 | | .66 | | -0.05 | | 0.08 |
|  | BI*DBC*PubDev | | | -0.01 | | 0.04 | | -0.29 | | .77 | | -0.09 | | 0.07 |
|  | BI*DBC*Sex | | | -0.02 | | 0.06 | | -0.30 | | .76 | | -0.14 | | 0.10 |
| **Outcome: Avoidance and Distress - General** | |  |  | |  | |  | |  | |  | |  |  |
|  | *Predictor* | | | *Coeff* | | *SE* | | *t* | | *p* | | *LLCI* | | *ULCI* |
| Step 1 | BI | | | 0.03 | | 0.02 | | 1.43 | | .16 | | -0.01 | | 0.08 |
|  | DBC | | | 2.14 | | 3.18 | | 0.67 | | .50 | | -4.18 | | 8.46 |
|  | Age | | | 4.27 | | 0.58 | | 7.40 | | **<.001** | | 3.12 | | 5.42 |
|  | Sex | | | -0.29 | | 1.23 | | -0.24 | | .81 | | -2.74 | | 2.15 |
|  | PubDev | | | -1.37 | | 0.98 | | -1.40 | | .17 | | -3.30 | | 0.57 |
| Step 2 | BI*DBC | | | -0.01 | | 0.03 | | -0.49 | | .63 | | -0.06 | | 0.04 |
|  | DBC*Age | | | -0.65 | | 1.04 | | 0.62 | | .53 | | -1.42 | | 2.72 |
|  | DBC*Sex | | | -1.06 | | 1.81 | | -0.59 | | .56 | | -4.66 | | 2.54 |
|  | DBC*PubDev | | | -0.12 | | 1.18 | | -0.10 | | .92 | | -2.46 | | 2.22 |
| Step 3 | DBC*Sex*Age | | | 1.03 | | 2.14 | | 0.48 | | .63 | | -3.22 | | 5.28 |
|  | DBC*Sex*PubDev | | | 0.66 | | 2.96 | | 0.22 | | .82 | | -5.21 | | 6.54 |
|  | BI*DBC*Age | | | 0.02 | | 0.03 | | 0.61 | | .54 | | -0.05 | | 0.09 |
|  | BI*DBC*PubDev | | | -0.03 | | 0.04 | | -0.77 | | .45 | | -0.12 | | 0.05 |
|  | BI*DBC*Sex | | | -0.08 | | 0.07 | | -1.13 | | .26 | | -0.21 | | 0.06 |

*Note*: Interaction terms were tested in individual models to avoid overcontrolling and maximize power; BI = behavioral inhibition; DBC delta-beta coupling; PubDev = pubertal development; Biological sex was coded as 1 = male, 2 = female.

**Figure S1**

**Scalp distribution of alpha power (lower activation indicates higher power).**


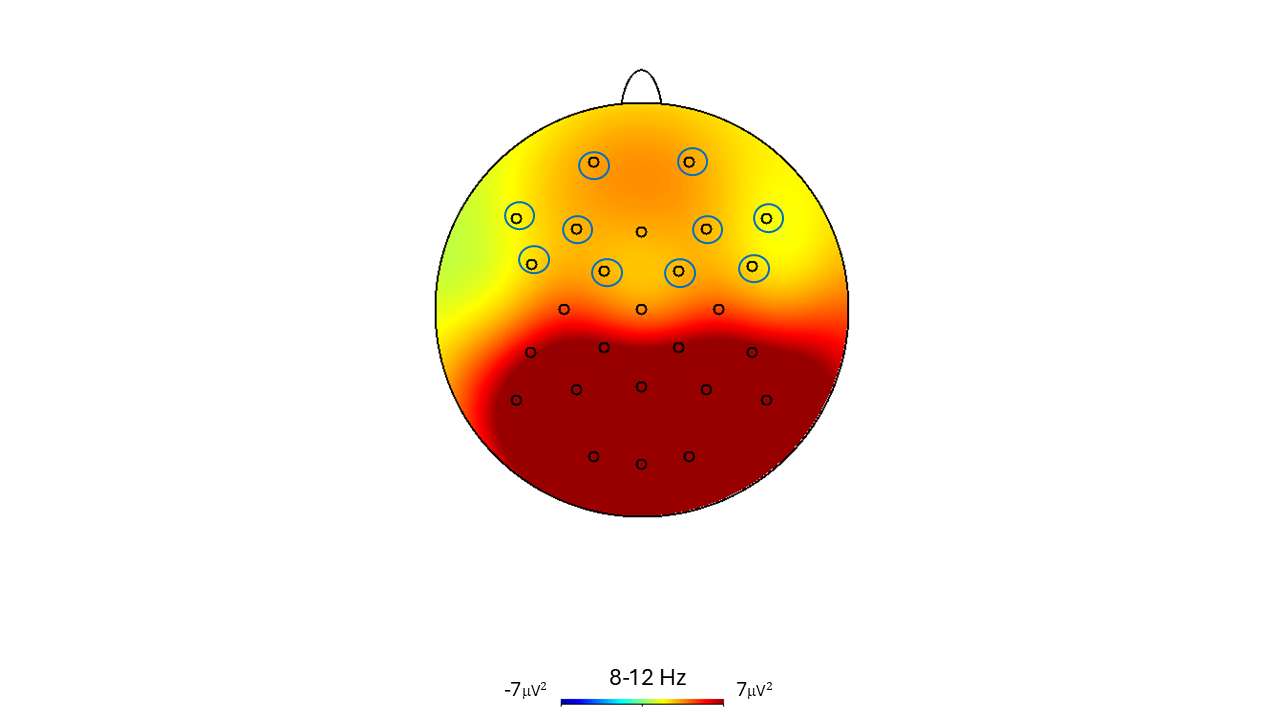


*Note:* Electrodes included in the frontal asymmetry clusters are circled in blue.
